# Proteomics of methyl jasmonate induced defense response in maize leaves against Asian corn borer

**DOI:** 10.1186/s12864-015-1363-1

**Published:** 2015-03-21

**Authors:** Yi Tong Zhang, Yu Liang Zhang, Si Xue Chen, Guo Hua Yin, Ze Zhong Yang, Samantha Lee, Chun Guang Liu, Dan Dan Zhao, Yu Kun Ma, Fu Qiang Song, Joan W Bennett, Feng Shan Yang

**Affiliations:** Key Laboratory of Molecular Biology of Heilongjiang Province, College of Life Sciences, Heilongjiang University, Harbin, 150080 China; Key Laboratory of Biology and Genetic Resources of Tropical Crops, Ministry of Agriculture, Institute of Tropical Bioscience and Biotechnology, Chinese Academy of Tropical Agricultural Sciences, Haikou, Hainan 571101 China; Majorbio Pharm Technology Co., Ltd., Shanghai, 201203 China; Department of Biology, Genetics Institute, Plant Molecular and Cellular Biology Program, Interdisciplinary Center for Biotechnology Research, University of Florida, Gainesville, Florida, 32610 USA; Department of Plant Biology and Pathology, Rutgers, The State University of New Jersey, New Brunswick, NJ 08901 USA; Institute of Pesticide Science, Hunan Agricultural University, Changsha, China; Engineering Research Center of Agricultural Microbiology Technology, Ministry of Education, Heilongjiang University, Harbin, 150500 China

**Keywords:** Maize, 2-DE, Mass spectrometry, Methyl jasmonate, qRT-PCR, Asian corn borer, Bio-control

## Abstract

**Background:**

Jasmonic acid (JA) and methyl jasmonate (MeJA) regulate plant development, resistance to stress, and insect attack by inducing specific gene expression. However, little is known about the mechanism of plant defense against herbivore attack at a protein level. Using a high-resolution 2-D gel, we identified 62 MeJA-responsive proteins and measured protein expression level changes.

**Results:**

Among these 62 proteins, 43 proteins levels were increased while 11 proteins were decreased. We also found eight proteins uniquely expressed in response to MeJA treatment. Data are available via ProteomeXchange with identifier PXD001793. The proteins identified in this study have important biological functions including photosynthesis and energy related proteins (38.4%), protein folding, degradation and regulated proteins (15.0%), stress and defense regulated proteins (11.7%), and redox-responsive proteins (8.3%). The expression levels of four important genes were determined by qRT-PCR analysis. The expression levels of these proteins did not correlate well with their translation levels. To test the defense functions of the differentially expressed proteins, expression vectors of four protein coding genes were constructed to express in-fusion proteins in *E. coli*. The expressed proteins were used to feed *Ostrinia furnacalis*, the Asian corn borer (ACB). Our results demonstrated that the recombinant proteins of pathogenesis-related protein 1 (PR1) and thioredoxin M-type, chloroplastic precursor (TRXM) showed the significant inhibition on the development of larvae and pupae.

**Conclusions:**

We found MeJA could not only induce plant defense mechanisms to insects, it also enhanced toxic protein production that potentially can be used for bio-control of ACB.

**Electronic supplementary material:**

The online version of this article (doi:10.1186/s12864-015-1363-1) contains supplementary material, which is available to authorized users.

## Background

The plant hormone jasmonic acid (JA) is involved in diverse developmental processes and defense responses to abiotic and biotic stresses. JA affects various stages of plant development including germination, root growth, tendril coiling, fertility, fruit ripening, tuberization, and senescence [1-4]. Methyl jasmonate (MeJA), the volatile form of JA, has been widely used to study jasmonate signaling pathways and mechanisms of plant defense. MeJA activates a signaling cascade of plant cell membrane genes. Following activation, the expression levels of defensive genes, such as proteinase inhibitors and pathogenesis-related (PR) proteins, change in order to regulate plant defense and immune responses [5].

With recent advances in genomic technologies, many jasmonate-responsive genes have been identified including those involved in jasmonate biosynthesis, secondary metabolism, and signal conduction and gene activation pathways [6]. MeJA-mediated signaling pathways and cellular responses can be researched using functional genomics and bioinformatics approaches. Most of the earlier studies examining the role of JA or MeJA in *Arabidopsis*, tomato, and tobacco employed microarray technology [7-10]. Studies have shown significant changes in the expression levels of induced genes. These studies have pushed the research toward the functional analysis of JA-response genes; however, due to the limitations of the microarray approach, the direct functions of JA-induced genes are unable to be determined.

The availability of genome sequences of many plant species and high-throughput technologies such as proteomics have facilitated a better understanding of the role of MeJA and its regulatory networks in plants. These later studies have been conducted in *Arabidopsis*, rice and wheat [11,12]. For example, the expression of JA-induced PR proteins and cellular protectant proteins in rice leaves were shown along with suppression of ribulose-1,5-bisphosphate carboxylase/oxygenase (RuBisCO), and exogenous application of JA induced the expression of several defense and stress-related genes [13,14]. Similarly, exogenous MeJA significantly enhanced disease resistance in wheat and showed significant increase of the expression of PR genes [15].

To date, there is limited research examining the effects of MeJA on signal pathways and cellular responses in maize. As a global crop, maize quality and yield is affected by environmental conditions and pathogen exposures. Previous studies report that exogenous application of MeJA induced physiological and molecular changes leading to increased resistance to pathogens and other stressors. Although these studies provide some insight to MeJA function in maize, there is a need to examine both the expression of genes and proteins since mRNA levels are not always consistent with protein levels due to post-transcriptional, translational, and post-translational regulatory activities. In order to adequately study the molecular basis of MeJA-induced changes in maize, we examined both gene and protein expressions. Recent proteomics studies in maize focus on drought [16], light [17], and temperature stresses [18]; however, MeJA responses in maize using proteomic approaches have not been reported.

The aim of the present study is to identify proteins and genes induced by MeJA and to determine how the protein levels are regulated by MeJA. Using proteomics and qRT-PCR technologies, we identified differentially expressed genes and proteins. We present the correlation between differentially expressed proteins and genes induced by MeJA involved in various cellular functions.

## Results

### Plant proteins under MeJA treatment

**Figure 1:**
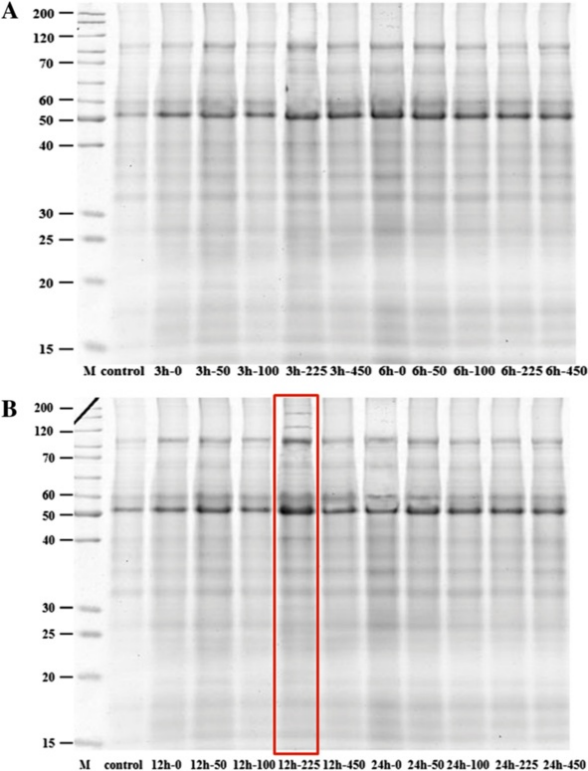
**SDS-PAGE of proteins extracted from maize leaves after MeJA treatment.** Lane M: Molecular weight marker; different lanes are labelled as treatment time (3 and 6 h in **A**; 12 and 24 h in **B)** and the concentrations of MeJA (0, 50, 100, 225 and 450 μM).

In this study, to determine the best induction conditions of exogenous application of MeJA, four time points (3, 6, 12, and 24 h) and four concentrations (50, 100, 225, and 450 μM) were tested. Total proteins of each group were analyzed using SDS-PAGE and the protein profiles were compared. The mass spectrometry proteomics data have been deposited to the ProteomeXchange Consortium [19] via the PRIDE partner repository with the dataset identifier PXD001793. The total protein concentration in maize reached the highest concentration at 225 μM of MeJA for 12 hours (Figure 1).

### The effects of feeding ACB larvae and adults with MeJA-treated maize

**Figure 2:**
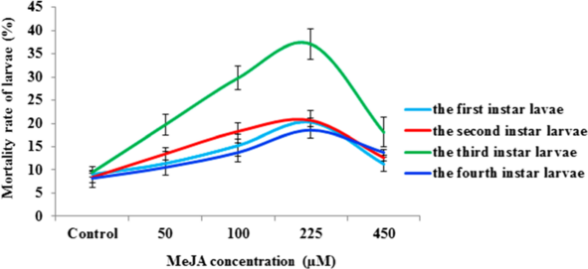
**Lethal effects of feeding Asian corn borer larvae with MeJA-treated maize leaves.**

To evaluate the effects of MeJA on different larval stages of *Ostrinia furnacalis*, commonly known as the Asian corn borer (ACB), we inspected the mortality rate of larvae fed on corn leaves treated with different concentrations of MeJA. Summarized in Figure 2, the mortality rates of larvae fed on leaves treated with different concentrations of MeJA were all higher than the controls. Increasing concentrations of MeJA caused the mortality rate of larvae to increase and reached its highest level at the concentration of 225 μM. There was a decrease in the mortality rate at the concentration of 450 μM. The 3^rd^ stage larvae were significantly affected by MeJA compared to the other stages of larvae (1^st^, 2^nd^, and 4^th^). Thus, we concluded that the ACB were significantly affected at 225 μM of MeJA (*P* < 0.05).

We also assayed the effects of MeJA on the developmental duration of different larval stages of the ACB. With increasing concentrations of MeJA, the number of days spent in different stages of development increased. Although 1^st^ instar larvae were not affected significantly, total duration of other stages of larvae (2^nd^, 3^rd^, and 4^th^) were significantly delayed by 225 and 450 μM of MeJA (*P* < 0.05). Compared to controls, 2^nd^, 3^rd^, and 4^th^ instar larvae exposed to 225 μM of MeJA were developmentally delayed by 24%, 22%, and 75%, respectively. The 5^th^ instar larvae were also significantly delayed at 100 and 225 μM of MeJA (*P* < 0.05) (Table 1). In summary, the differences between the treated and control groups were significant at 225 μM of MeJA for 12 hours.

**Table 1:** **Effects of different concentrations MeJA treatment on the developmental duration of different instar Asian corn borer larvae**

**MeJA (μM)**	**Developmental duration of different stages of larvae (Days)**					
	**1** ^**st**^ **instar**	**2** ^**nd**^ **instar**	**3** ^**rd**^ **instar**	**4** ^**th**^ **instar**	**5** ^**th**^ **instar**	**Total duration**
Control	4.17 ± 0.08a	3.78 ± 0.11b	3.96 ± 0.03c	1.99 ± 0.05b	4.25 ± 0.14b	18.15 ± 0.34c
50	4.33 ± 0.33a	3.89 ± 0.11b	3.97 ± 0.12c	1.93 ± 0.23b	4.50 ± 0.29b	18.62 ± 0.85c
100	4.27 ± 0.15a	3.97 ± 0.03b	4.33 ± 0.09bc	2.17 ± 0.09b	4.87 ± 0.09a	19.60 ± 0.31bc
225	4.83 ± 0.17a	4.67 ± 0.17a	4.83 ± 0.12a	3.50 ± 0.25a	5.72 ± 0.43a	23.56 ± 0.24a
450	4.33 ± 0.33a	4.33 ± 0.33a	4.07 ± 0.07c	3.29 ± 0.20a	4.56 ± 0.29b	20.58 ± 0.20b

Notes: The data are average ± standard deviation. Different letters indicate the significant differences between treatments at *P* < 0.05 (Duncan’s multiple range test).

The adult life span and average number of eggs produced by the female per day also were significantly affected by MeJA. The adult life span of the treatment group was shortened by 0.23 ~ 1.33 days compared to control group. There was a decrease in the number of eggs with increased concentrations of MeJA with a significant decrease in the number of eggs produced by female at 100, 225, and 450 μM (Table 2).

**Table 2:** **Effects of different concentrations of MeJA on the life span and fecundity of adult Asian corn borers**

**MeJA (μM)**	**Adult life span (days)**	**No. of eggs laid per female per day**	**Adult fecundity**
Control	9.00 ± 0.58a	22.20 ± 0.81a	110.78 ± 0.83a
50	8.77 ± 0.15ab	20.97 ± 0.55a	100.72 ± 0.70b
100	8.07 ± 0.12abc	19.27 ± 0.27b	77.5 ± 0.46c
225	7.67 ± 0.28c	18.60 ± 0.16b	70.89 ± 1.83d
450	7.80 ± 0.06bc	19.14 ± 0.21b	74.76 ± 1.11c

Notes: The data are average ± standard deviation. Different letters indicate the significant differences between treatments at *P* < 0.05 (Duncan’s multiple range test).

**Figure 3:**
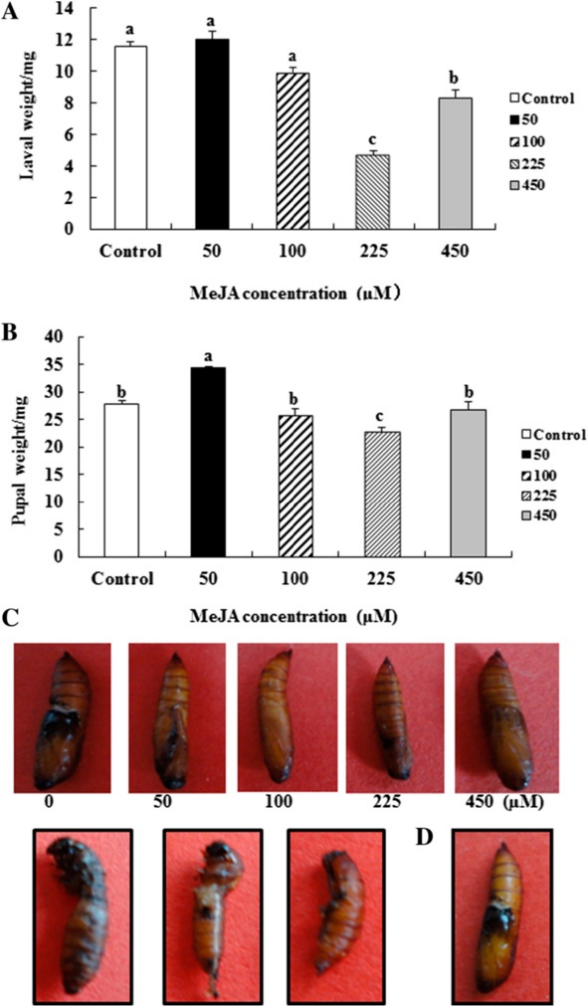
**Effects of MeJA-induced maize leaves on the growth and development of ACB.** Larval **(A)** or pupal **(B)** weight (mg) after MeJA treatment; abnormal **(C)** and normal **(D)** pupae after MeJA treatment.

We examined the effects on insect growth and development of ACB larvae fed on maize treated with different concentrations of exogenous MeJA. The growth and development of ACB larvae were significantly inhibited with MeJA. Compared to the control group, the weight of larvae treated with 225 and 450 μM of MeJA was significantly decreased by 3.29 ~ 6.86 mg (28.3% ~ 59.1%), and the weight of pupae significantly decreased (*P* < 0.05) by 4.87 mg (Figure 3AB). Many of the pupae fed by maize leaves treated by 225 μM of MeJA for 12 hours were abnormal (Figure 3C).

We also evaluated the effects of MeJA on the life table of ACB. Compared to the control group, When ACB larvae/adults were fed corn leaves treated with 225 μM of MeJA, the net reproductive decreased 55.6%, the intrinsic rate of increase decreased 30.8%, and time to double the population increased 44.4% (Table 3).

**Table 3:** **Effects of 225 μM of MeJA on the life table parameters of Asian corn borer**

**Treatment**	**Net reproductive rate**	**Mean generation time**	**Intrinsic rate of increase**	**Finite rate of increase**	**Double population time (days)**
Control	14.81	37.18	0.0725	1.0752	9.56
MeJA	6.57	37.47	0.0502	1.0515	13.80

### The proteome profile of maize leaves in response to MeJA and its functional classification

**Figure 4:**
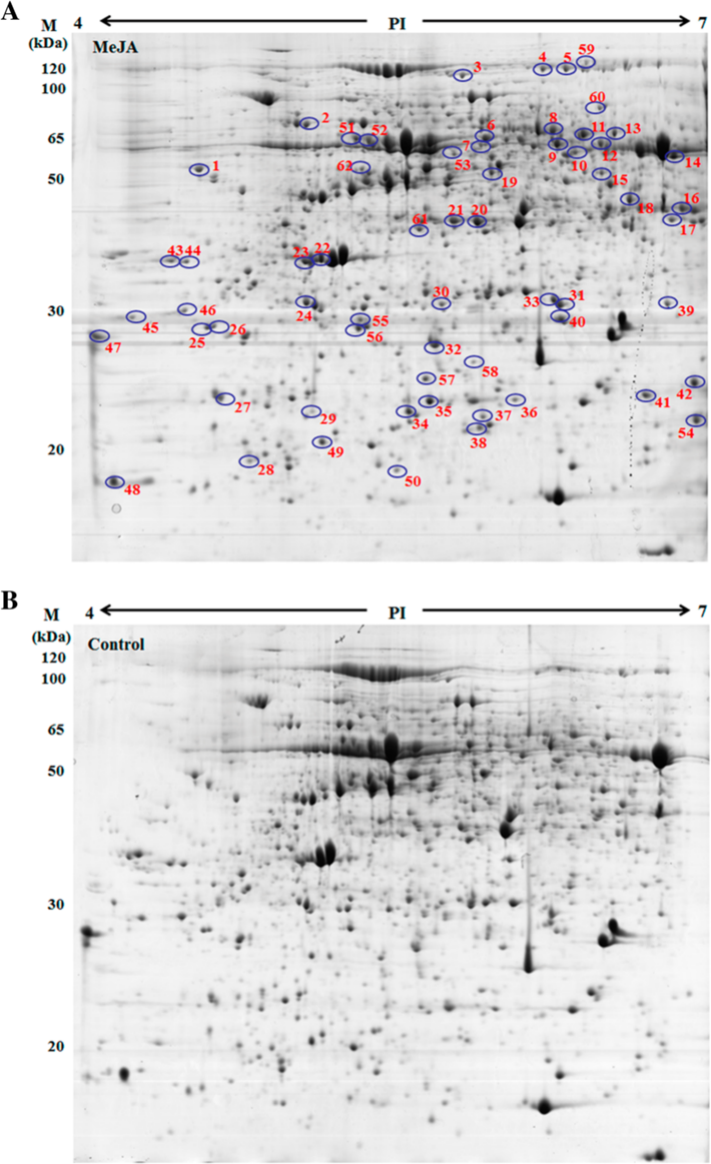
**2-D gel analysis of proteins extracted from maize leaves treated by 225 μM MeJA for 12 h.** A total of 1000 μg of protein was loaded on each IPG strip (PI 4–7). Protein spots were visualized using Coomassie brilliant blue staining. Protein spots from both MeJA treatment **(A)** and control group **(B)** were identified.

2-D gels were performed to separate proteins in two dimensions for quantitative analysis and protein identification. Quantitative image analysis by the ImageMaster™ 2-D Platinum 7.0 revealed significant changes in 62 protein spots (Figure 4 and Additional file 1: Table S1) with a change greater than 1.5 fold (*P* < 0.05). Of the 62 MeJA-responsive proteins, 43 proteins were significantly increased, 11 were decreased, and 8 were detectable. Protein spots from both control and MeJA group were excised and identified (Additional file 2: Table S2).

**Figure 5:**
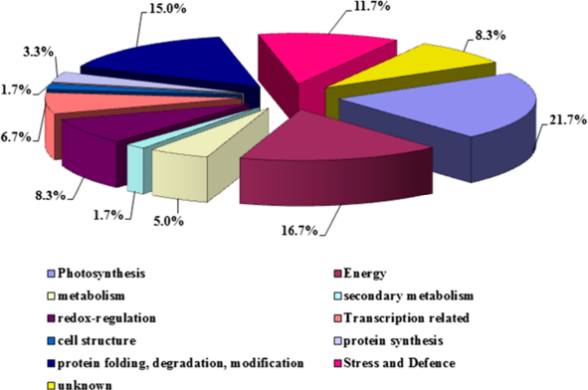
**Functional classifications of differentially expressed proteins in response to MeJA treatment.** The pie chart shows the distribution of the MeJA-responsive proteins into their functional classes in percentages.

The identified proteins further were classified based on their subcellular localization and biological process according to annotations in the Swiss-Prot database. All of the identified proteins were classified into 10 functional groups, covering a wide range of pathways and functions: photosynthesis (21.7%), energy (16.7%), protein folding, degradation, modification (15.0%), stress and defense (11.7%), redox-regulation (8.3%), transcription related protein (6.7%), metabolism (5.0%), protein synthesis (3.3%), secondary metabolism (1.7%), and cell structure (1.7%). We also found some unknown proteins (8.3%). The four largest groups of proteins, consisting of 39 proteins, were associated with photosynthesis, energy, protein folding and defense-related proteins, which indicated that multiple cellular processes are important for plant defense responses triggered by MeJA (Figure 5).

Five heat shock-related proteins/chaperones were responsive to the MeJA treatment. Seven spots (spots 8, 11, 13, 31, 33, 34, and 49) were increased in the presence of MeJA. Spots 8, 11, and 13 were identified to be beta-D-glucosidase precursor. Spots 31 and 33 were identified to be glutathione transferases (GSTs) 5 and 19, respectively. The pathogenesis-related protein 1 was also found to respond to MeJA in this study. We found increases in two superoxide dismutase (SODs), SOD [Cu-Zn] 2 (spot 36) and SOD [Mn] 3, 4, mitochondrial precursor (spot 40). We identified GDP-mannose 3, 5-epimerase. Spot 30 was identified as dehydroascorbatereductase (DHAR) and increased after MeJA treatment. Four proteins involved in plant metabolism (spots 17, 22, 26, and 37) were increased by MeJA treatment. Spots 17, 22, and 26 play major roles in detoxification in plant secondary metabolism. Spot 17, identified as aldo-ketoreductase family 1, member B1, has frequently been implicated in the metabolism of exogenous and endogenous toxicants, including those stimulated by stresses [20,21]. Spot 22, identified as inorganic pyrophosphatase, was increased after MeJA treatment. MeJA treatment induced the differential expression of four translation-related spots (spots 10, 43, 44, and 50) in leaves. Spot 10 was identified as plasminogen activator inhibitor 1 RNA-binding protein and increased by MeJA treatment. Spots 43 and 44 were decreased and identified as nucleic acid binding protein 1 and ribonucleoprotein, respectively. Spot 14 was identified as hypothetical protein LOC100194135, containing an agglutinin domain and increased in response to MeJA treatment (Figure 4).

### Quantitative RT-PCR analysis of four defense genes

**Figure 6:**
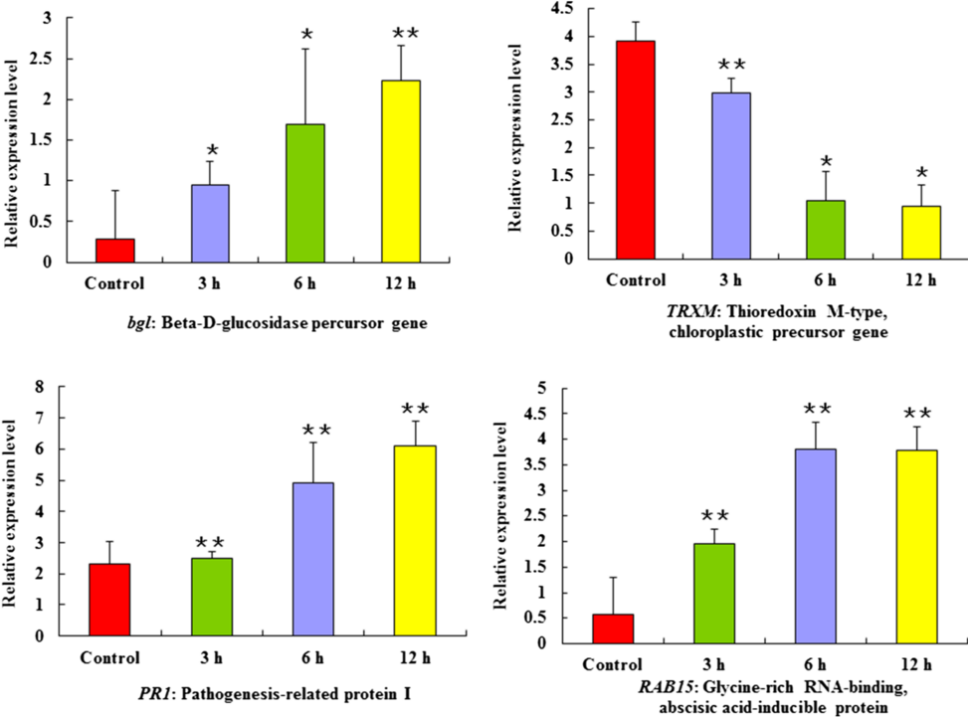
**Quantitative RT-PCR analysis of four defense genes was performed after MeJA treatment.** The chart shows the expression abundance of the selected genes’ transcripts in response to MeJA treatment, * and ** indicate that values are significantly different at *P* < 0.05 and *P* < 0.01 level, respectively.

Four genes closely associated with plant defense were selected for qRT-PCR analysis: beta-D-glucosidase precursor gene (*bgl*); thioredoxin M-type, chloroplastic precursor gene (*TRXM*); pathogenesis-related protein 1 gene (*PR1*); and glycine-rich RNA-binding, abscisic acid-inducible protein gene (*RAB15*). The primers of these four genes and the reference gene (*actin*) are listed in Table 4. The qRT-PCR results are shown in Figure 6. The primers were designed using Primer Premier 5.0. The suitable restriction enzymes were added when we designed the primers for in-fusion defensive proteins. Following MeJA treatment, there was more than 1.5 fold increase in the expression levels of these four genes. The *bgl* (GenBank No. HQ834242), *PR1* (GenBank No. HQ834244), and *RAB15* (GenBank No. HQ834245) genes were up-regulated while *TRXM* (GenBank No. HQ834243) was down-regulated after MeJA induction.

**Table 4:** **Primers of four defense genes**

**Genes**	**Expressed level**	**Primers for qRT-PCR**	**Primers for expression vectors**
***Actin***	-	S- 5′ CGGCAGCCTCCATACCAA 3′	-
		A - 5′ GCCAAGAACAGCTCCTCA 3′	-
***bgl***	Up	S- 5′ TCGCCACAAAGCAGTAAGC 3′	S- 5′gc**GAGCTC**ATGGCTCCACTTCTCGCCGCAG3′
		A- 5′ ACCAAAGATGAAGTCAGAGGG 3′	A - 5′cg**CTCGAG**AGCTGGCGTAATAATCTTCTTG3′
***TRXM***	Up	S- 5′ TTGGTGATGGCGTGCGAGAC 3′	S- 5′gc**GGATCC**ATGGCCATGGAGACGTGCTT3′
		A- 5′ TGGATGCCGTAGGCGTCGT 3′	A - 5′cg**AAGCTT**TGAGCTACCGATGTACTTGT3′
***PR1***	Up	S- 5′ TCGCACATCAAGGTGGAGC 3′	S- 5′gc**GGATCC**ATGGCCTCCGTCAACAGCT3′
		A- 5′ ATGGTTTAGTTGTAGGCGTCGG 3′	A - 5′cg**AAGCTT**GTTGTAGGCGTCGGGGTT3′
***RAB15***	Down	S- 5′ GAGAATGCCTTCGCCTCCTAC 3′	S- 5′ gc**GGATCC**ATGGCGGCGGCTGATGTGGAGT3′
		A- 5′ GTCGAGCTCCTTGCCGTT 3′	A - 5′cg**AAGCTT**GTCCCTCCAGCCGCCGCCG3′

Notes: The bold sequences are restriction enzyme sites. S: upstream primers; A: downstream primers.

### Cloning and expression of four fusion proteins and evaluation of their effects on ACB

**Figure 7:**
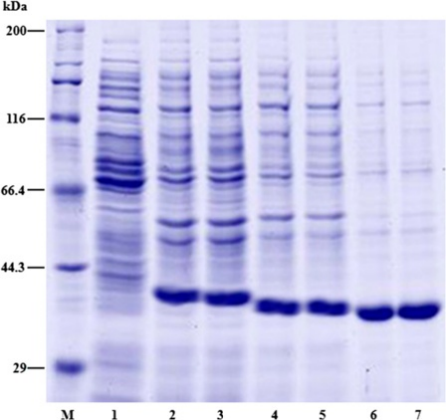
**SDS-PAGE of recombinant proteins expressed in** ***E*** **.** ***coli*** **of BL21(DE3).** Lane M: Molecular weight marker; lane 1: the proteins extracted from BL21(DE3) transformed with empty vector as control; lanes 2–7: the recombinant proteins from BL21 (DE3) transformed with pET28a-TRXM, pET28a-RAB15, and pET28a-PR1 induced with 0.1 mM and 1 mM IPTG, respectively.

**Figure 8:**
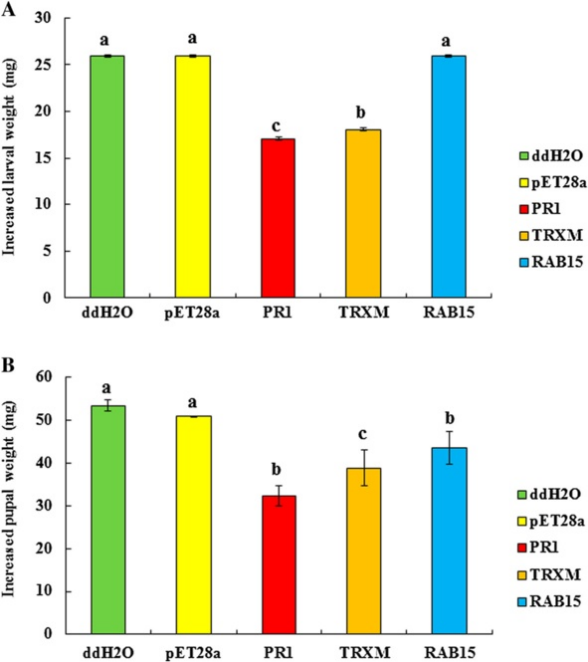
**Effects of recombinant proteins on the larval and pupal weight of Asian corn borer.** Increased larval **(A)** and pupal **(B)** weight were measured.

Genes *TRXM*, *RAB15*, *PR1*, and *bgl* were cloned and the PCR products were subjected to 1% agarose electrophoresis shown in Additional file 3: Figure S2. The positive clones were screened by enzyme digestion and PCR amplification (Additional file 4: Figure S3). The induced proteins and the control were loaded on a 5% SDS-PAGE, and three fusion proteins were successfully expressed (Figure 7). The concentrations of the purified proteins PR1, TRXM, and RAB15 were tested as 1.14, 1.03, and 1.50 mg/mL by Bradford assay. We fed ACB with the same concentration of purified protein. Compared to ddH_2_O and pET-28a empty vector groups, fusion proteins of TRXM and PR1 significantly delayed the growth and development of ACB, and the inhibition rates were 30.3% and 34.1%, respectively. The fusion protein of RAB15 did not affect the weight of larvae but significantly affected the weight of the ACB pupae (Figure 8).

### Comparative analysis of transcriptome data and proteome data

Comparing proteome data with the transcriptome data, we identified 23 genes out of the 137 genes that were matched to 26 proteins by identity or high homology (Additional file 2: Table S2). When the relative protein expression levels were compared with mRNA levels, 14 displayed similar expression trends, and 13 showed opposite trends of expression. For example, spot 50 (RAB15 protein) showed decreased protein level after induction by MeJA, while qRT-PCR showed increased level of mRNA expression. For those that followed similar up or down expression trends at the mRNA and protein levels, they differed in fold change at the two levels.

### A hypothetical model of MeJA response in maize

**Figure 9:**
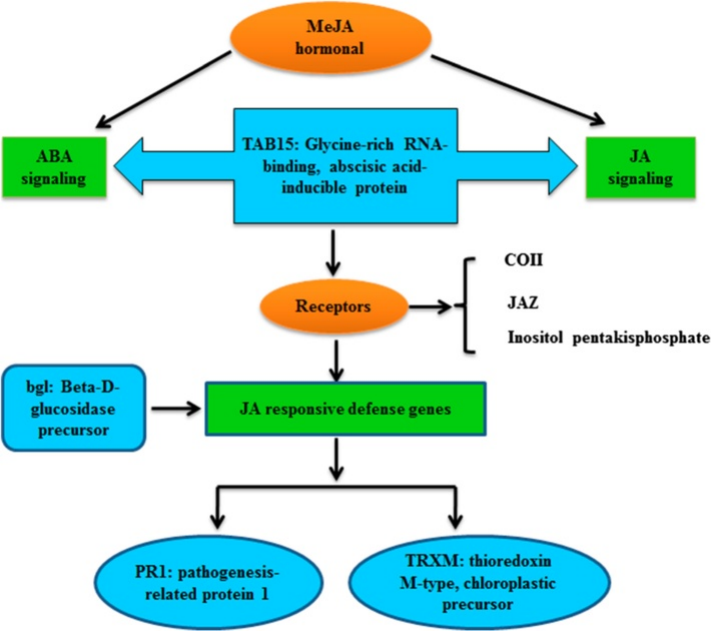
**Hypothetical model of MeJA response in maize.**

In Figure 9, we offer a possible pathway that describes how MeJA may induce responsive proteins with insect defense capabilities. In the hypothetical model, TAB15 is viewed as central to ABA and JA signaling, and it transfers the signal to receptors to induce the expression of JA responsive defense genes for PR1- or TRXM-mediated resistant reactions. BGL protein also may involve this JA responsive defense. Thus, maize could perceive the MeJA signal to regulate transcription, protein synthesis, and related bioprocesses, thereby affecting the levels of functional proteins to defend against pathogens such as ACB. These processes work cooperatively to establish a defensive state in maize plants under attack by pathogenic herbivores.

## Discussion

### Two-dimensional gel electrophoresis

While 2-D gels provide information about protein quantity, charge, and mass of an intact protein, the method has limitations when analyzing of multiple proteins present in a single spot. Specifically, the change of the spot intensity caused by experimental treatment usually cannot be assigned to a particular protein [22]. In our study, spot 39 showed a significant 1.5-fold increase after MeJA treatment. The two proteins identified in this spot were glutathione transferase III (a) and the hypothetical protein LOC100191154 containing a proteasome subunit alpha domain (Additional file 1: Table S1). Since the 2-D gel method has an advantage in distinguishing proteins of closely related family members and proteins with different modifications leading to changes in pI and molecular weight, the pattern of these protein spots on the gel may indicate possible genetic isoforms and/or post-translational modifications PTMs. The possible existence of different protein isoforms is worthy of further investigation.

Under herbivore attack, secondary metabolites such as phenolic acids, tannins, and alkaloids are produced to induce and enhance plant defense. In addition to secondary metabolites, the plant defense response is mediated by defense proteins [23]. The proteins induced by exogenous MeJA are considered to be an important “defense weapon” in plants. For example, plant protease inhibitors (PIs) induced by wounding bind protease enzymes in the insect, thereby decreasing or inhibiting protein digestion activities in the gut. The expression of PIs is regulated by a JA pathway [24]. The expression of the JA-inducible proteins (JIPs), threonine deaminase enzyme and arginase, enhance plant tolerance to herbivores [25,26]. The plant response to exogenous MeJA includes massive changes in physiology and biochemistry. The proteins involved in JA synthesis, regulation of cell wall structures, stress and defense responses, and photosynthesis are all induced by MeJA [27]. In this study, we have provided additional candidates for the MeJA-responsive proteome.

### Some important proteins in plant defense reactions

#### Protein folding, degradation, modification-related proteins

Heat-shock proteins (Hsps)/chaperones are responsible for protein folding, assembly, translocation, and degradation in normal cellular processes. They stabilize proteins and membranes, and assist in biotic and abiotic stress tolerance by protein refolding [28]. Thus, the ubiquitous Hsps/chaperone system plays pivotal roles in cells under normal and stressful conditions [29]. Here we identified five heat shock-related proteins/chaperones in maize response to the MeJA treatment that also increased in abundance after MeJA treatment (Additional file 1: Table S1). As low-molecular-mass Hsps, small Hsps (sHsps, 12–40 kDa) form a widespread and more diverse family than other Hsps/chaperones with respect to sequence similarity, cellular location and functions [30-32]. The increased abundance of sHsps/chaperones after MeJA treatment suggests that Hsps are synthesized in plant cells in response to biotic stresses. In addition, S-phase kinase-associated-protein 1 (SKP1), was shown to be a component of a SCF (SKP1-Cullin-F-box) complex which is necessary for ubiquitin-mediated protein degradation in eukaryotes [33]. In *Arabidopsis*, many F-box proteins are found to interact with ASK1 or ASK2, which suggests that they could form various SCF complexes, and that SCF complexes may regulate plant development by affecting the signal pathways of auxin, gibberellin, and ethylene [34]. In our study, the SKP1-like protein 1A increased in response to MeJA treatment in maize (Additional file 1: Table S1) which indicates that this protein may mediate the MeJA signaling pathway to enhance plant defense responses [34].

### Defense and stress-related proteins

Plant responses to abiotic and biotic stresses involve the expression of a large number of proteins, many of which are believed to be crucial components of the plant’s self-defense mechanism. In this study, seven spots (spots 8, 11, 13, 31, 33, 34, and 49) increased in the presence of MeJA. Spots 8, 11 and 13 were identified to be a beta-D-glucosidase precursor. Beta-glucosidases play significant roles in diverse aspects of plant physiology and activate chemical defense compounds that function as a chemical deterrent to herbivore and pathogen attack [35]. In some cases, a functional diversification of glutathione transferases (GSTs) has roles in isomerization, reduction, and the binding, protection, and transport of secondary products [36]. In this study, spots 31 and 33 were identified to be GSTs 5 and 19, respectively. Plant GSTs have been associated with responses to biotic and abiotic stress, hormones and developmental changes. Our results suggest that GSTs mediate plant defense through direct and indirect methods. We also detected the pathogenesis-related protein 1 as responsive to MeJA. This response has been observed previously in *Arabidopsis*, rice, and tobacco with exogenous application of JA [7-10].

### Redox-related proteins

To reduce oxidative injuries induced from reactive oxygen species (ROS), plants have developed enzymatic systems for scavenging ROS [37]. Superoxide dismutase (SOD) catalyzes the conversion of the toxic O_2_^−^ radial to oxygen (O_2_) and H_2_O_2_. Catalase, peroxidase, and ascorbate peroxidase (APX) can convert ROS such as H_2_O_2_ to water and O_2_. ROS work together as enzymatic antioxidant defense systems in yeasts, animals, and plants. We found an increase of two SODs (spots 36 and 40). They were SOD [Cu-Zn] 2 (spot 36) and SOD [Mn] 3,4, mitochondrial precursor (spot 40). Initially discovered as regulators of light-dependent malate biosynthesis in the chloroplast [38,39], plant thioredoxins are involved in a large panel of reactions related to metabolism, defense, and development [40].

We also identified GDP-mannose 3,5-epimerase. This enzyme catalyzes a reversible epimerization of GDP-D-mannose that precedes the committed step in the biosynthesis of vitamin C, an enzyme cofactor and an antioxidant [41]. It plays a crucial role in response to induction by MeJA in many essential physiological processes such as biosynthesis of the cell wall, phytohormones, secondary metabolites, cell division, growth, stress resistance, and photoprotection in plants [42]. Spot 30 was identified as dehydroascorbatereductase (DHAR) and increased after MeJA treatment. Expression of DHAR responsible for regenerating ascorbic acid from an oxidized state regulates the cellular redox state, which in turn affects cell response and tolerance to environmental ROS. Ascorbate is essential for the detoxification of environmental toxins and products of oxidative stress [43].

### Metabolism-related proteins

In *Zea mays*, four proteins involved in plant metabolism (spots 17, 22, 26, and 37) were increased by MeJA treatment (Additional file 1: Table S1 and Figure 4). Spots 17, 22, and 26 play major roles in detoxification in plant secondary metabolism. Spot 17, identified as aldo-ketoreductase family 1, member B1, has frequently been implicated in the metabolism of exogenous and endogenous toxicants, including those stimulated by stresses [20,21]. Spot 22, identified as inorganic pyrophosphatase, increased after MeJA treatment. Pyrophosphoric acid in plant tissues is mainly produced in the biosynthesis of RNA, protein, carbohydrates, etc. Accumulation of pyrophosphoric acid can inhibit normal biosynthesis and affect plant growth. In plant cells, inorganic pyrophosphatase can decompose pyrophosphoric acid into inorganic pyrophosphate to detoxify and enable plant growth. On the other hand, some related studies showed that the protein coding genes were induced by ABA suggesting ABA signal conduction [44]. We deduce that the inorganic pyrophosphatase may be induced by MeJA treatment, and through the MeJA signal pathway, regulate its related gene expression.

Compared with other studies that many metabolites such as carbohydrates, lipids, protein metabolite related proteins, and secondary metabolism, related proteins induced by MeJA, we only identified a few proteins related to metabolism. This may be closely related to the treatment time and concentration of MeJA.

RNA-binding proteins (RBPs) play key roles in post-transcriptional control of RNAs, which along with transcriptional regulation, are an important way to regulate patterns of gene expression during development [45]. Nucleic acid-binding protein (NBP) belongs to a family of nuclear-encoded chloroplast proteins, which share a common domain structure and are thought to be involved in the post-transcriptional regulation of chloroplast gene expression [46]. Post-transcriptional regulation can occur at many different steps in RNA metabolism, including splicing, polyadenylation, mRNA stability, mRNA localization and translation. Our results suggest that JA treatment can regulate transcription and post-transcription of RNAs. Most importantly, we identified a glycine-rich, RNA-binding, ABA-inducible protein with decreased abundance in response to MeJA. This family of proteins is involved in the regulation of post-transcriptional gene expression processes including pre-mRNA splicing, mRNA transport, mRNA stability and translation [47-49]. It has been reported that the glycine-rich, RNA-binding, ABA-inducible protein 7 expression is modulated via a circadian clock and by a variety of abiotic and biotic stress conditions [50]. Recently, this protein was shown to affect stomata movement in response to abiotic stress, and thus, played a role in freezing tolerance and response to dehydration under high salinity stress [51]. The glycine-rich, RNA-binding, abscisic acid-inducible protein has 82% positive homology with a protein from rice associated with disease resistance [52]. Except for complex light harvesting, proteins, microarray studies did not identify significant expression changes in genes of the other proteins, highlighting the importance of proteomic studies. Interestingly, microarray analysis showed that MeJA treatment led to remarkably decreased transcripts of ABA-responsive genes, indicating that an antagonistic interaction occurs between the JA and ABA signaling pathways in abiotic stress responses [53].

### Cell structure

Spot 14, which increased response to MeJA treatment, was identified as the hypothetical protein LOC100194135 containing an agglutinin domain. It is involved in the regulation of gene expression in stressed plants through specific protein-carbohydrate interactions with regulatory cytoplasmic/nuclear glycoproteins. Many flowering plants contain sequences encoding putative homologues of the tobacco lectin-JA inducible proteins (JIPs), which imply a possible ubiquitous family of lectins with a specific endogenous role [54]. However, there is no report regarding the function of LOC100194135 and whether it belongs to JIPs. It is possible that further analysis of this protein may enhance our understanding of JIPs.

In this study, we confirmed the presence of known MeJA responsive proteins, such as GSTs, pathogenesis-related protein 1, thioredoxin M-type (chloroplastic precursor), and a beta-D-glucosidase precursor in addition to identifying new stress and defense related proteins such as chaperone protein ClpB 1, putative, expressed and putative small heat shock protein, 5′-partial and so forth. The proteins play different specific biological functions, e.g., some play major roles in plant response to stresses. At present, information about these protein functions is not complete, but their expression changes after MeJA induction will provide valuable references for us to elucidate the induced defense mechanisms.

### Comparative analysis of transcriptome data and proteome data

The only available transcriptomics analysis of leaves treated by MeJA was carried out using microarrays in *A. thaliana* and in rice. Although 137 jasmonate-responsive genes were differentially expressed in *A. thaliana* [53], the study did not thoroughly identify the gene functions in the JA or MeJA signaling pathways.

We also found that changes of beta-D-glucosidase precursor gene and pathogenesis-related protein 1 gene were similar at the transcriptional level and the protein level. Thioredoxin M-type, chloro-plastic precursor, and glycine-rich RNA-binding, abscisic acid-inducible protein exhibited the most drastic changes at the protein level, but their expression levels were not similar to their protein abundances. The mRNA levels are not always consistent with protein levels because of various post-transcriptional, translational, and post-translational levels regulatory mechanisms [20-23].

It should also be noted that the molecular weight of fusion proteins was much higher than predicted and may be attributed to the expression vector. Some studies have shown that the His-tag strong electric charge may influence many other His-tag fusion proteins to form higher apparent molecular weight in SDS-PAGE [55].

## Conclusions

In summary, we applied a 2-D gel approach to identify the proteome changes in maize in response to MeJA treatment. Proteomics analyses identified several proteins involved in stress and defense responses. These proteins may serve important roles in MeJA signal transduction by MS/MS mapping. Further analysis by qRT-PCR showed that the mRNA transcription levels did not necessarily correlate with the abundance of their corresponding proteins and highlights the importance of conducting both proteomics and transcriptome analyses.

## Methods

### Ethics statement

The study and protocols for collection of proteomic data and procedures were approved by all the authors’ institutional and/or licensing committee, and we confirmed that all experiments were performed in accordance with relevant guidelines and regulations (Editor: Dr. Leonard Foster, University of British Columbia, Canada).

### Chemicals and reagents

All the chemicals and solvents were of analytical grade. All the reagents were used without further purification. Bradford assay (Coomassie blue protein assay) was from Applygen Technologies Inc. (Beijing, China).

### Plant growth and MeJA treatment

Seeds of *Zea mays* L. Dongnong 250 were used in this study. The seeds were sown into plastic basins (10 cm × 15 cm) and kept at room temperature. The plants were grown at 27 ± 1°C with a 14:10 hour (light:dark) photoperiod with 80% relative humidity. When the maize plants developed to the interior leaf period, they were randomized into 20 groups and control groups (10 basin/each group). Four time intervals (3, 6, 12, and 24 hours) and four concentrations (50, 100, 225, and 450 μM of MeJA) were selected to treat the leaves of corn. One batch of leaves was frozen in liquid nitrogen and stored at −80°C for protein extraction, while the others were used for the biological activity assay on ACB. Three batches of biological replicates were collected for both control and treated samples.

### Effects of maize treated with MeJA on ACB

The corn borers were maintained in the chamber at 25 ± 2°C under the light:dark (14:10) and the relative humidity was kept at 70 ~ 80%. The artificial diets were made according to the method of Zhou *et al*. [56].

To evaluate the effects of feeding ACB with corn leaves treated with different concentration of MeJA, we selected similar size larvae kept in a glass petri dish (90 × 15 mm). For 1^st^ and 2^nd^ instar larvae, 30 were used for each treatment. For 3^rd^ and 4^th^ instar larvae, 20 were used for each treatment. The experiments were repeated at least three times. The development of all stages, weight, mortality rate, and pupation rate were recorded every day. Adults were transferred into a wide mouth bottle after emergence from pupae, fed on a cotton ball soaked with 10% sucrose water, and the fecundity was recorded every day until death.

### Effects on larval/pupal weight

The 3^rd^ instar larvae were selected and kept for 12 h without food. Then, they were fed with corn leaves with or without MeJA treatment. For each experiment, 30 larvae were used and the experiment was repeated three times. Larval mass was recorded after three days, weighing was continued until the larvae pupated.

### Effects on the life table of ACB

Pupae were fed with corn leaves treated with 225 μM of MeJA. Then 20 unmated females/males (female:male = 1:1) were selected, transferred to a wide mouth bottle, and fed with 10% sucrose water. The fecundity and the adult life span (days) were recorded until all the adults died. ACB eggs were transferred to fresh corn leaves and the developmental durations, the hatching rate, the larvae duration, the pupae duration, and the adult life, etc. were recorded to construct the life table of ACB [57]. Experiments were repeated in triplicate.

### Protein extraction, 2-D gel, image and nanoESI MS/MS analysis

After MeJA treatment, corn leaves were grounded into powder in liquid nitrogen. The protein was precipitated in a 10% TCA, cold acetone solution (w/v) containing 0.07% (v/v) β-mercaptoethanol at −20°C for two hours. After centrifugation at 40,000 × g at 4°C for one hour, the supernatant was discarded and the pellet was rinsed with −20°C cold acetone containing 0.07% (v/v) β-mercaptoethanol. The final pellet was vacuum-dried and solubilized in 3 mL of 7 M (w/v) urea containing 2 M (w/v) thiourea, 40 mM DTT, and 1% (v/v) protease inhibitor mixture (GE Healthcare, USA) on ice for about one hour. Insoluble material was removed by centrifugation at 100,000 rpm for one hour. The protein concentration was determined using the 2-D Quant kit (GE Healthcare, USA) with BSA as a standard. Samples were frozen in liquid nitrogen and stored at −80°C for further analysis.

The proteins from the control group and MeJA-treated plants were compared by using 2-D gel image analysis. The isoelectric points (pI) of the spots ranged from 4 to 7, and the molecular mass ranged from 10 to 120 kDa. For each sample, 1 mg total protein in 450 μL rehydration buffer (7 M urea, 2 M thiourea, 2% CHAPS, 0.5% IPG buffer with PH 4–7, and 0.04 M DTT) was loaded onto a 24 cm, pH 4–7 linear gradient IPG strip (GE Healthcare, USA). Isoelectric focusing was performed using an Ettan IPGphor 3 isoelectric focusing system according to the manufacturer’s instruction. The focusing conditions were as follows: active rehydration was carried out at low voltage liquefied for 12 h, followed by 300 V for 1 h, 600 V for 1 h, 1000 V for 1 h, with a linear increase of voltage to 8,000 V for 12 h at 20°C. The voltage was held at 10,000 V until the total voltage hours reached 80,000. After IEF, the strips were equilibrated with an equilibration solution (50 mM Tris with pH 8.8, 6 M urea, 30% glycerol, 1% DTT, and 2% SDS) followed by 2.5% iodoacetamide in the equilibration solution, each for 15 min. The second dimension was performed on 12.5% polyacrylamide gels using an Ettan DALT Six Electrophoresis Unit (GE Healthcare, USA) according to the manufacturer’s instructions. The 2-D gel electrophoresis experiments were repeated three times using protein samples prepared independently from MeJA treated and control maize.

Proteins were visualized by Coomassie brilliant blue R250 and gel images were acquired using an ImageScanner (GE Healthcare, USA). Replicate gels from control and MeJA treatment were analyzed with ImageMaster 2-D Platinum Software Version 7.0 (Amersham Biosciences, USA). Experimental molecular weight (kDa) of each protein was estimated by comparison with the protein markers, and experimental isoelectric points were determined by its migration on the IPG strip. The abundance of each protein spot was estimated by the percentage volume (%vol). Only those spots with significant and reproducible changes were considered to be differentially expressed proteins. The normalized volumes of the spots from replicate gels were subjected to student’s ANOVA test (*P* < 0.05) and only statistically significant data were considered. Protein in-gel tryptic digestion and nanoESI MS/MS analysis were carried out on a QSTAR XL MS/MS system (AB Sciex Inc., USA) as previously described [58]. The peptide MS/MS spectra were searched against an NCBI non-redundant fasta database (8,224,370 entries, June 20, 2014) using Mascot search engine (http://www.matrixscience.com). Mascot was set up to search green plants only, assume trypsin digestion and one allowed miscleavage. The mass tolerance for both parent ion and fragment ion mass was set to be 0.2 Da. Iodoacetamide derivatization of Cys, deamidation of Asn and Gln, and oxidation of Met were specified as variable modifications. Unambiguous identification was judged by the number of peptides, sequence coverage, Mascot score and the quality of MS/MS spectra (Additional file 5: Figure S1) [58].

### qRT-PCR

Maize leaves treated by MeJA for different periods (6 and 12 h) and the control leaves were harvested. Total RNA was extracted using Invitrogen kit and reverse transcribed with a PrimeScript RT Reagent kit (TaKaRa, Japan) according to the manufacturers’ instructions. qRT-PCR assays were performed using ABI7500 (Applied Biosystems, USA) and *actin* (GenBank accession number X97726) was used as an internal standard gene. Diluted aliquots of the reverse transcribed cDNAs were used as templates in RT-PCRs containing the SYBR Green PCR Master Mix (SYBR Green Real Time PCR Kit, HaiGene, China). Primers are listed in Table 4. qRT-PCRs were performed with an initial activation step of the DNA polymerase at 94°C for 5 min, followed by 40 cycles of 94°C for 12 s, 58°C for 30 s, 72°C for 40 s, 79°C for 1 s, and a step of plate reading. Triplicate reactions were carried out for each sample to ensure reproducibility. Negative controls only contained gene-specific primer pairs. At the end of each PCR program, a melting curve was generated and analyzed with Dissociation Curves Software (ABI 7500 Software v2.0, USA). The length and specificity of PCR products were verified by agarose gel electrophoresis. Gene expression was quantified using the comparative cycle threshold (Ct) method [59-61].

### Cloning and expression of four fusion proteins and evaluation of their effects on corn borer

To identify the *TRXM*, *TAB15*, *PR1*, and *bgl* genes in maize, total RNA was extracted from the leaves using TRIzol regent (Invitrogen, USA) and RT-PCR was performed using a PrimeScript RT Reagent kit (TaKaRa, Japan) according to the respective manufacturer’s instructions. The full-length coding regions of these four genes’ cDNA were amplified by PCR using gene-specific primers containing *Nde*I and *Hind*III, or *Sac*I and *Xho*I restriction enzyme sites (Table 4). The amplified fragments were inserted into pMD19-T vector (TaKaRa, Japan) and transformed into *Escherichia coli* DH5α, and then cultured in LB liquid medium in a rotary shaker for 12–16 h. The cells were harvested by centrifugation and used for plasmid extraction. Full-length cDNA sequences of these four genes were confirmed by restriction digestion and DNA sequencing.

The amplified four genes were digested with the restriction enzymes (*Nde*I and *Hind*III, or *Sac*I and *Xho*I), and ligated into pET-28a doubly digested with the same enzymes. The recombinant plasmids were named as pET28a-TRXM, pET28a-RAB15, pET28a-PR1, and pET28a-BGL, which could produce the TRXM, RAB15, PR1, and BGL fusion protein, respectively. The correct expression vectors were verified by diagnostic restriction digestion and DNA sequencing. The correct plasmids were then transformed into *E. coli* strain of BL21 (DE3), and the bacteria were grown overnight at 37°C in 100 ml LB broth with 100 μg/mL ampicillin. The overnight culture was inoculated to one liter of fresh LB medium and was grown at 28°C with shaking at 250 rpm. When the OD_600_ of the culture reached 0.6, 0.1 mM IPTG was added to induce the expression of protein and then made the culture grown at 25°C. After an additional four to five hour cultivation, the cells were harvested by centrifugation at 6000 × g for 10 min. The bacterial pellets were resuspended in 10 ml of cell lysis buffer (25 mM Tris, 200 mM NaCl, pH 8.0, 1 mM PMSF) and were lysed by sonication and centrifuged at 15000 × g for 10 min. The supernatant was collected. His-tagged proteins were purified under native condition with His spinTrap columns as described in the manual (GE Healthcare, USA). The columns were equilibrated with PBS binding buffer with 20 mM imidazole. The samples were loaded at a concentration of 750 mg/mL. The columns were washed using PBS washing buffer with 100 mM imidazole for five times. The in-fusion proteins were eluted using PBS elution buffer with 500 mM imidazole.

The 3^rd^ instar larvae of ACB were selected to evaluate the effects of fusion proteins on their growth and development. The feeding assay was performed after the larvae were starved for 12 hour with 3 replications and 20 larval in each replicate. The empty vector and ddH_2_O were used as controls. The artificial diets mixed with recombinant proteins were replenished daily. Food consumption, movement and general morphology of larvae were recorded daily. After seven days, the weight of larvae and pupae were measured. The feeding assays were continued until all the larvae became pupae. Then the weight of pupae was measured.

### Data analysis

All the data were analyzed by SPSS17.0 statistical software (SPSS Inc., Chicago, IL, USA).

## Additional files

Additional file 1:
**Detailed information of comparative analysis of microarray data and proteome data by homology BLAST.**
Additional file 2:
**Detailed information of identified proteins and their relative expression levels in** ***Zea*** **mays.**
Additional file 3:
**Clones of the four defense genes.** Lane M_2_: DL2000 DNA marker, lanes 1–4: PCR amplification of four defense genes (*TRXM*, ~ 507 bp; *RAB15*, ~ 470 bp; *PR1*, ~ 482 bp; *bgl*, ~ 1692 bp). Additional file 4:
**Identification of the recombination plasmids.** A: Lane M_1_: DL15000 DNA marker; lane M_2_: DL2000 DNA marker; lanes 1 and 4: single digestion of the recombination plasmids of pET28a-PR1 and pET28a-RAB15, respectively; lanes 2 and 3: double digestion of the recombination plasmids of PET28a-PR1 and pET28a-RAB15. B: lane 1: the plasmid of pET28a; lane 2: single digestion of the recombinant plasmid of pET28a-BGL; lane 3: double digestion of the recombinant plasmid of pET28a-BGL. Additional file 5:
**MS/MS spectra of two peptides.** A beta-D-glucosidase (A) and a pathogenesis-related protein 1 (B) were identified from *Zea mays*. The b and y ion series were manually inspected, and the ion scores and the ranking in the Mascot search were considered.

## Abbreviations

JA: Jasmonic acid
MeJA: Methyl jasmonate
ACB: Asian corn borer
2-D gel: Two dimensional gel electrophoresis
pI: Isoelectric points
RuBisCO: Ribulose-1, 5-bisphosphate carboxylase/oxygenase
*bgl*: beta-D-glucosidase precursor gene
*TRXM*: Thioredoxin M-type, chloroplastic precursor gene
*PR1*: Pathogenesis-related protein 1 gene
*RAB15*: Glycine-rich RNA-binding, abscisic acid-inducible protein gene
IPG: Immobilized pH gradient
GST: Glutathione transferases
SCF: SKP1-Cullin-F-box
SKP1: S-phase kinase-associated-protein 1
ROS: Reactive oxygen species
SOD: Superoxide dismutase
APX: Ascorbate peroxidase
DHAR: Dehydroascorbate reductase
RBPs: RNA-binding proteins
NBP: Nucleic acid-binding protein
JIPs: JA inducible proteins
