# Supplementary figures and images for: Proteomics of methyl jasmonate induced defense response in maize leaves against Asian corn borer
Source: BMC Genomics. 2015 Mar 21;16(1):224. doi: 10.1186/s12864-015-1363-1 (PMC4375847; doi:10.1186/s12864-015-1363-1)

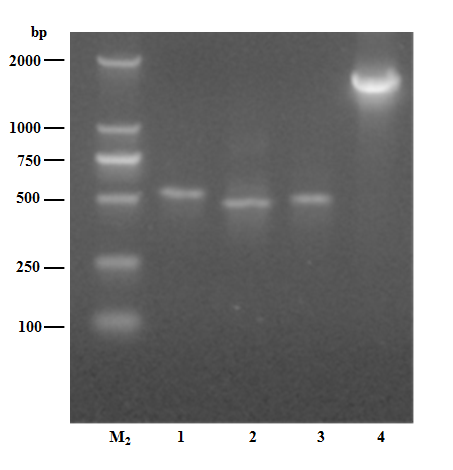

Supplement: Additional file 3: — Clones of the four defense genes. Lane M2: DL2000 DNA marker, lanes 1–4: PCR amplification of four defense genes (TRXM, ~ 507 bp; RAB15, ~ 470 bp; PR1, ~ 482 bp; bgl, ~ 1692 bp). [file 12864_2015_1363_MOESM3_ESM.tiff]

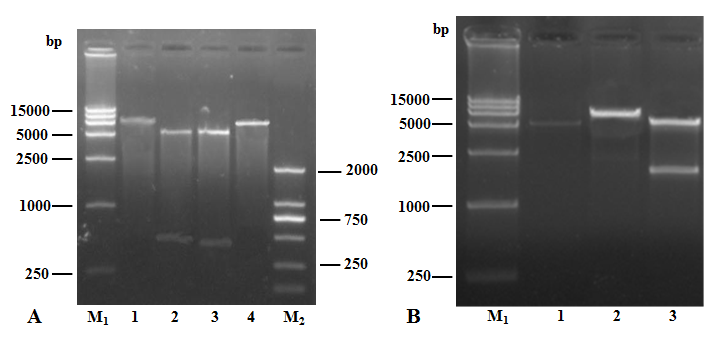

Supplement: Additional file 4: — Identification of the recombination plasmids. A: Lane M1: DL15000 DNA marker; lane M2: DL2000 DNA marker; lanes 1 and 4: single digestion of the recombination plasmids of pET28a-PR1 and pET28a-RAB15, respectively; lanes 2 and 3: double digestion of the recombination plasmids of PET28a-PR1 and pET28a-RAB15. B: lane 1: the plasmid of pET28a; lane 2: single digestion of the recombinant plasmid of pET28a-BGL; lane 3: double digestion of the recombinant plasmid of pET28a-BGL. [file 12864_2015_1363_MOESM4_ESM.tiff]

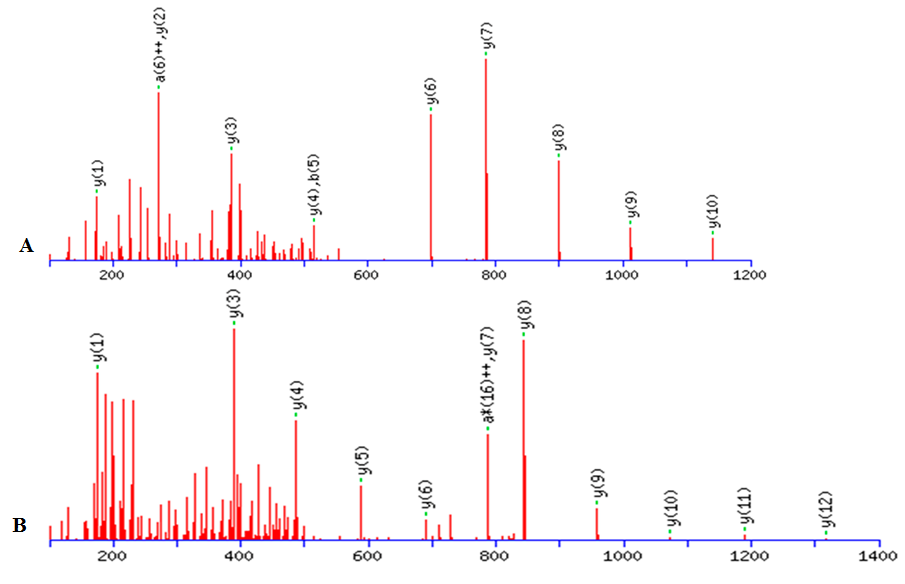

Supplement: Additional file 5: — MS/MS spectra of two peptides. A beta-D-glucosidase (A) and a pathogenesis-related protein 1 (B) were identified from Zea mays. The b and y ion series were manually inspected, and the ion scores and the ranking in the Mascot search were considered. [file 12864_2015_1363_MOESM5_ESM.tiff]
